# Global reaction to the recent outbreaks of Zika virus: Insights from a Big Data analysis

**DOI:** 10.1371/journal.pone.0185263

**Published:** 2017-09-21

**Authors:** Nicola Luigi Bragazzi, Cristiano Alicino, Cecilia Trucchi, Chiara Paganino, Ilaria Barberis, Mariano Martini, Laura Sticchi, Eugen Trinka, Francesco Brigo, Filippo Ansaldi, Giancarlo Icardi, Andrea Orsi

**Affiliations:** 1 Department of Health Sciences, University of Genoa, Genoa, Italy; 2 Hygiene Unit,”Ospedale Policlinico San Martino IRCCS” teaching hospital, Genoa, Italy; 3 Department of Neurology, Christian Doppler Klinik, Paracelsus Medical University, Center for Cognitive Neuroscience, Salzburg, Austria; 4 Department of Public Health Technology Assessment, UMIT—University for Health Sciences, Medical Informatics and Technology, Hall i.T., Innsbruck, Austria; 5 Department of Neurology, Franz Tappeiner Hospital, Merano, Italy; 6 Department of Neurological, Biomedical, and Movement Sciences, University of Verona, Verona, Italy; New York City Department of Health and Mental Hygiene, UNITED STATES

## Abstract

**Objective:**

The recent spreading of Zika virus represents an emerging global health threat. As such, it is attracting public interest worldwide, generating a great amount of related Internet searches and social media interactions. The aim of this research was to understand Zika-related digital behavior throughout the epidemic spreading and to assess its consistence with real-world epidemiological data, using a behavioral informatics and analytics approach.

**Methods:**

In this study, the global web-interest and reaction to the recently occurred outbreaks of the Zika Virus were analyzed in terms of tweets and Google Trends (GT), Google News, YouTube, and Wikipedia search queries. These data streams were mined from 1^st^ January 2004 to 31^st^ October 2016, with a focus on the period November 2015—October 2016. This analysis was complemented with the use of epidemiological data. Spearman’s correlation was performed to correlate all Zika-related data. Moreover, a multivariate regression was performed using Zika-related search queries as a dependent variable, and epidemiological data, number of inhabitants in 2015 and Human Development Index as predictor variables.

**Results:**

Overall 3,864,395 tweets, 284,903 accesses to Wikipedia pages dedicated to the Zika virus were analyzed during the study period. All web-data sources showed that the main spike of researches and interactions occurred in February 2016 with a second peak in August 2016. All novel data streams-related activities increased markedly during the epidemic period with respect to pre-epidemic period when no web activity was detected. Correlations between data from all these web platforms resulted very high and statistically significant. The countries in which web searches were particularly concentrated are mainly from Central and South Americas. The majority of queries concerned the symptoms of the Zika virus, its vector of transmission, and its possible effect to babies, including microcephaly. No statistically significant correlation was found between novel data streams and global real-world epidemiological data. At country level, a correlation between the digital interest towards the Zika virus and Zika incidence rate or microcephaly cases has been detected.

**Conclusions:**

An increasing public interest and reaction to the current Zika virus outbreak was documented by all web-data sources and a similar pattern of web reactions has been detected. The public opinion seems to be particularly worried by the alert of teratogenicity of the Zika virus. Stakeholders and health authorities could usefully exploited these internet tools for collecting the concerns of public opinion and reply to them, disseminating key information.

## Introduction

The striking diffusion of Zika virus, an emerging arthropod-borne flavivirus transmitted by day-time active mosquitoes, especially *Aedes* species, represents an emerging global health threat [1]. Zika was incidentally discovered in 1947 in Uganda among primates, meanwhile the first human cases were registered in 1952 in Uganda and Tanzania. Since the 60-80s, human infections have occasionally occurred across Asia and Africa, although typically mild and with limited symptoms [2]. The first major human outbreak occurred in 2007 in the island of Yap, Federal States of Micronesia. In October 2013 the Zika virus was again responsible of a large outbreak in French Polynesia [3,4].

Since May 2015, when the first human cases were reported in Brazil, Zika virus has spread rapidly, in particular throughout South America, Central America and Caribbean. To date, 69 countries and territories, most of them located in Central and South America, have reported continuing mosquito-borne transmission of the virus with thousands of suspected Zika virus cases, and in 12 countries the person-to-person transmission of Zika virus, probably via sexual route, have been documented [5].

The disease generated by Zika virus is usually mild with fever, skin rash and conjunctivitis reported as the most frequent symptoms [6,7], even though Zika virus infection have been recently associated with Guillain-Barré syndrome [8]. Nonetheless, the main concerns about the virus regard the link between prenatal Zika virus infection and adverse birth outcomes, most notably microcephaly and other serious brain anomalies [9]. Recently, researchers from the US Centers for Disease Control and Prevention (CDC) have estimated that maternal infection with the Zika virus in the first trimester of pregnancy was associated with an estimated 1% to 13% risk of microcephaly [10].

It has been recently shown that global health threat attract public interest, as in the case of the recent outbreak of Ebola [11]. Since the Internet and the Web represent the main source of easily-accessible health-related information in many parts of the world, the global interest about Zika virus has generated a great amount of related Internet searches [12]. Moreover, social media interaction tend to increase in response to news coverage, especially for novel health threat [13,14].

Novel data streams, including internet search data and social media, such as Google searches, Google Plus, Facebook and Twitter posts, as well as Wikipedia access logs, are the only source for real-time insights into behavioral medicine, a multidisciplinary field incorporating medicine, social science and public health [15–18]. Concerning infectious diseases, novel data streams enable to capture and assess features of epidemic dynamics, such as public engagement, interest, behaviors perceptions and misinformation, that cannot be tracked and monitored by classical surveillance approaches, thus adding value to the traditional epidemiological techniques [19–23]. With respect to the current Zika epidemic, some studies have investigated the public reaction to Zika in terms of web searches and social media mentions over limited time periods [24–26]. In this view, we aimed to analyze and interpret the global and local interest and reaction to the recently occurred outbreaks of the Zika Virus, evaluated in terms of GT, Google News, YouTube, and Wikipedia search queries and tweets, for terms related to the Zika virus infection, in the period from 1^st^ January 2004 to 31^st^ October 2016, with a focus in the period from 1^st^ October 2015 to 31^st^ October 2016. In such a way, we endeavored to better understand the complex link between epidemiological changes in disease incidence, clinical changes in disease severity and symptoms, changes in media coverage and the time-varying novel data streams-related usage pattern. In particular, our aim was to understand Zika-related digital behavior throughout the epidemic spreading and to assess its consistence with real-world epidemiological data. This research question has practical implications and benefit in that health-care institutions and providers can exploit internet search data and social network interaction in order to explore the public reaction to infectious diseases outbreak, to identify disease-related information and to the provide real-time and more effective messages, giving information, news update, promoting events, and calling for preparedness.

## Material and methods

### Novel data streams analysis

Consistently with our research question in the current study, five different novel data streams were exploited, using a massive data mining approach and with a special focus on GT. Each data source was purposively selected to provide insights on a different level of analysis or specificity, in that each novel data stream captures a particular segment of the population and a particular behavior and reaction to Zika epidemic outbreak. Table 1 summarizes the various data sources described in the following sections. For all websites exploited for the collection of data, we complied with the terms of service.

**Table 1:** Novel data streams used for capturing public reaction to Zika epidemic outbreak.

Novel data streams	Description	Availability of collected data	Availability of processed data	Purpose
Google Trends	It enables to monitor and track Internet searches, by country	From 1^st^ January 2004 to 31^st^ October 2016	Monthly and yearly basis	To investigate the impact of the Zika epidemic breakout on health-information seeking behavior
Google News	It enables to monitor and track news coverage.	From 1^st^ January 2008 to 31^st^ October 2016	Weekly and monthly basis	To assess the impact of the Zika epidemic outbreak on news dissemination and consumption
YouTube	It enables to monitor and track specific video upload and consumption	From 1^st^ January 2008 to 31^st^ October 2016	Weekly and monthly basis	To investigate the impact of Zika epidemic outbreak on video upload and consumption
Wikitrends	It enables to count Wikipedia page visits and accesses, by language.	From December 2007 to January 2016	Weekly and monthly basis	To evaluate the impact of the Zika epidemic outbreak on specific information retrieval
Twitter			Weekly and monthly basis	To assess the impact of the Zika epidemic outbreak on social media usage and, in particular, on Tweet production

#### Google Trends

GT (Google Inc, Menlo Park, CA, USA) is a well-established, freely available, web-based tracking system that enables a visualization of hit-search volumes throughout the years, from 2004 onwards. GT was previously known as Google Insights for Search and provided the users with a visualization of the locations in terms of flux volumes for a given keyword. It was closed and subsequently merged to GT in 2012. GT provides a relative search volume (RSV), which is computed as the percentage of queries concerning a particular term for a given location and time period. This figure is, then, normalized by the highest query share of that term over the time series and presented on a scale from 0 to 100. Each point of the graph generated by GT is divided by the highest point, which is conventionally set at 100.

GT was used to explore Internet activity related to Zika Virus. The study period was from 1^st^ January 2004 to 31^st^ October 2016, since data before 2004 were not available, with a focus on the last year (from 1^st^ November 2015 to 31^st^ October 2016), when Google searches for Zika virus-related information have remarkably increased.

GT enables to search using two different search strategies, namely the “search term” and the “search topic” options. In the first case, the exact string of terms provided by the user is searched, whilst in the second case not only the typed term(s) but all those related to the provided keyword(s) are searched. Usually, using the second search strategy results into a broader search.

We used five different terms: “Zika” as search term, “Zika Virus” as both search term and search topic, “ZIKV” as search term and “Zika Virus disease” as search term, since it is possible to search for up to five queries each time [27].

Furthermore, we analyzed at global level the spatial localization of web search queries and the top searches and rising searches related to the search term or search topic. Specifically, top searches are terms that are most frequently searched with the term entered in the same search session, within the chosen category, country, or region; rising searches are terms that were searched for with the term entered, which had the most significant growth in volume in the requested time period. Assessing the top searches and the rising searches allows to describe the main topics of queries related to the above-mentioned search terms used for the research.

Finally, from 1^st^ November 2015 to 31^st^ October 2016 we descriptively analyzed the changes in the web search query, for the keyword that yielded the greatest RSV, at the global level and in all countries where autochthonous mosquito-borne Zika virus infections have been reported. Moreover, we compared these data with web search reactions in countries with possible endemic transmission or evidence of local mosquito-borne Zika infections in 2016, in countries with evidence of local mosquito-borne Zika infections in or before 2015, but without documentation of cases in 2016, or outbreak terminated, in countries with evidence of person-to-person transmission of Zika virus, other than mosquito-borne transmission, in order to compare when the highest peak of RSV occurred. Cumulatively, 80 countries were analyzed.

#### Twitter

Twitter (available at https://twitter.com) is an online microblogging service launched in March 2006, which enables its users to freely post and read short 140-character messages known as "tweets". It was mined since inception to 31 October 2016, using “zika” as a key-word; weekly and monthly number of Zika-related tweets was extracted.

#### Wikipedia

Wikitrends (freely available at www.wikipediatrends.com) and Wikipedia article traffic statistics (freely accessible at http://stats.grok.se/) are valuable open-source tools that enable scholars to quantitatively assess the absolute number of accesses to a given Wikipedia page. They were mined, using “zika” as a key-word, from December 2007 to January 2016 (when the latest data are available) in all languages. Data were extracted as daily data and aggregated on a weekly and monthly basis.

#### YouTube

YouTube (freely accessible at https://www.youtube.com) enable users to upload video material, as well as to download it. The number of normalized downloads of material related to the Zika virus, searched using “zika” as a key-word, was recorded from 1^st^ January 2008 to 31^st^ October 2016. Data were available on a weekly and monthly basis.

#### Google News

Google News (freely available at https://news.google.com/) is an aggregator of worldwide news. The number of normalized consumed news related to Zika and searched using “zika” as a key-word was recorded from 1^st^ January 2008 to 31^st^ October 2016. Data were available on a weekly and monthly basis.

### Epidemiological data

Epidemiological data about Zika were retrieved from the World Health Organization (WHO) and from the Pan American Health Organization (PAHO) [5,28]. In particular, we extracted weekly confirmed cases at global level, and cumulative suspected and confirmed Zika cases, imported cases, cases of microcephaly, and Zika incidence rate for each country belonging to PAHO at 3^rd^ November 2016.

### Statistical analysis

Before commencing any statistical analysis, data were visually inspected for outliers and checked for normality. Spearman’s correlation was performed to correlate all Zika-related data retrieved from the used novel data streams (namely, GT, Twitter, Google News, Wikipedia and YouTube). Further, Spearman’s correlation was used to correlate GT-generated data and epidemiological data at country level. Moreover, Spearman’s correlation was performed to correlate the number of weekly cases of Zika and search queries volumes from GT, Google News, and YouTube.

A generalized multivariate regression model was performed using Zika-related RSV for each country belonging to PAHO as a dependent variable, and epidemiological data (cumulative suspected and confirmed Zika cases, imported cases, cases of microcephaly, and Zika incidence), number of inhabitants in 2015 and Human Development Index (HDI) as predictor variables. The HDI was retrieved from the official website of the United Nations Development Programme, available at http://report.hdr.undp.org/ (last accessed on 2 December 2016). The HDI is a composite statistical indicator, which combines life expectancy, education and income indices as proxies of country development. Residual analysis was performed in order to confirm the normal distribution of data.

Computation was done using the Statistical Package for Social Science (SPSS) software v21.0.0 (IBM Corporation, Armonk, NY, US). Values ≤0.05 were considered statistically significant. All data on the number of Zika cases and the GT index are available as supplementary material.

## Results

**Fig 1:**
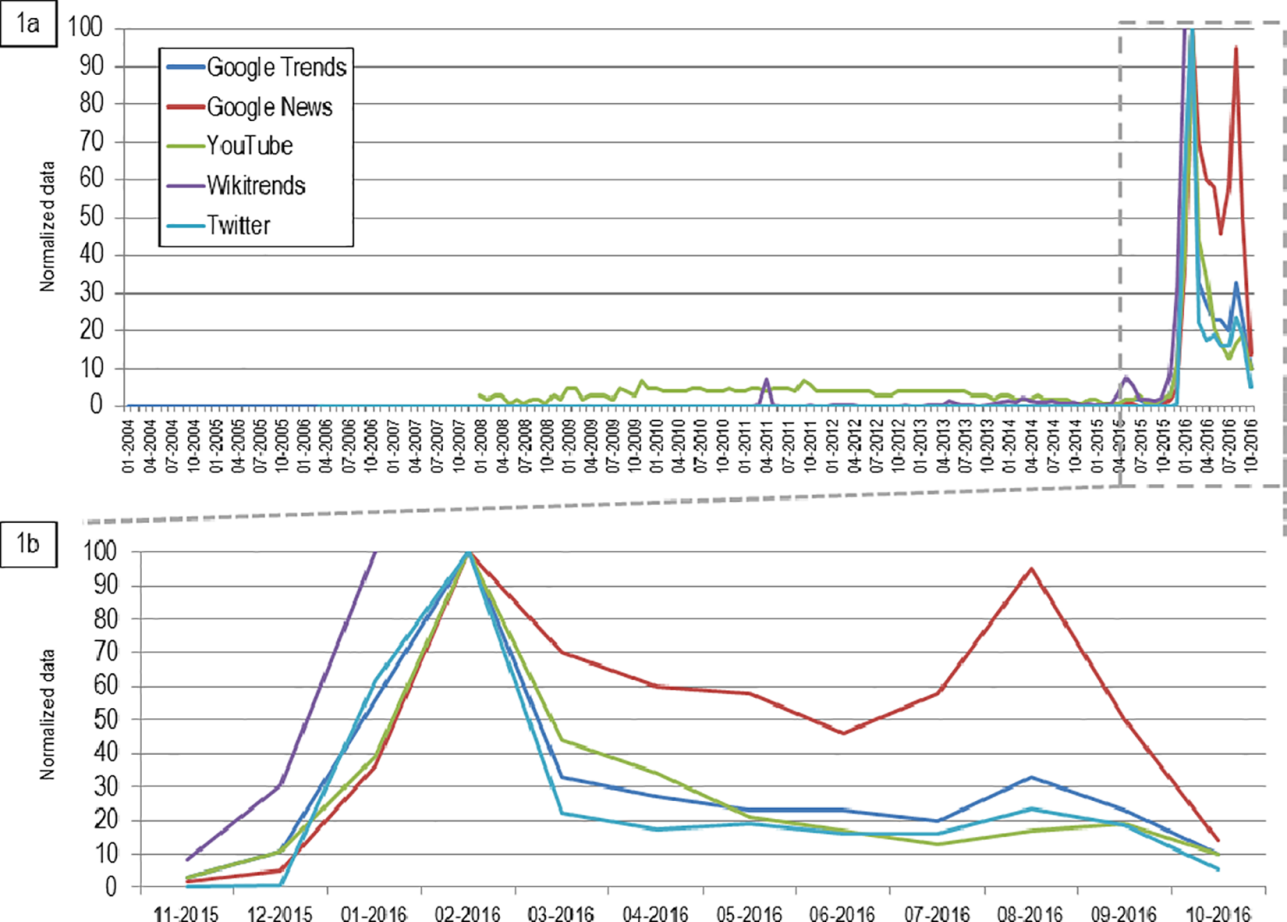
**Monthly normalized data of web-searches and social network interactions related to Zika retrieved from Google Trends, Google News, Wikitrends, YouTube and Twitter from January 2004 to June 2016 (1a) and from November 2015 to October 2016 (1b)**.

In Fig 1, all monthly data of web-searches and social network interactions related to Zika retrieved from the novel data streams analyzed in this study were reported. From January 2004 to November 2015, the normalized interactions from all data sources remained under 10. All data sources registered the peak of interactions in February 2016. Notably, Google News reported another peak of interest in August 2016, corresponding to an increase in searches or interactions trough the other web platforms.

### Google Trends

**Fig 2:**
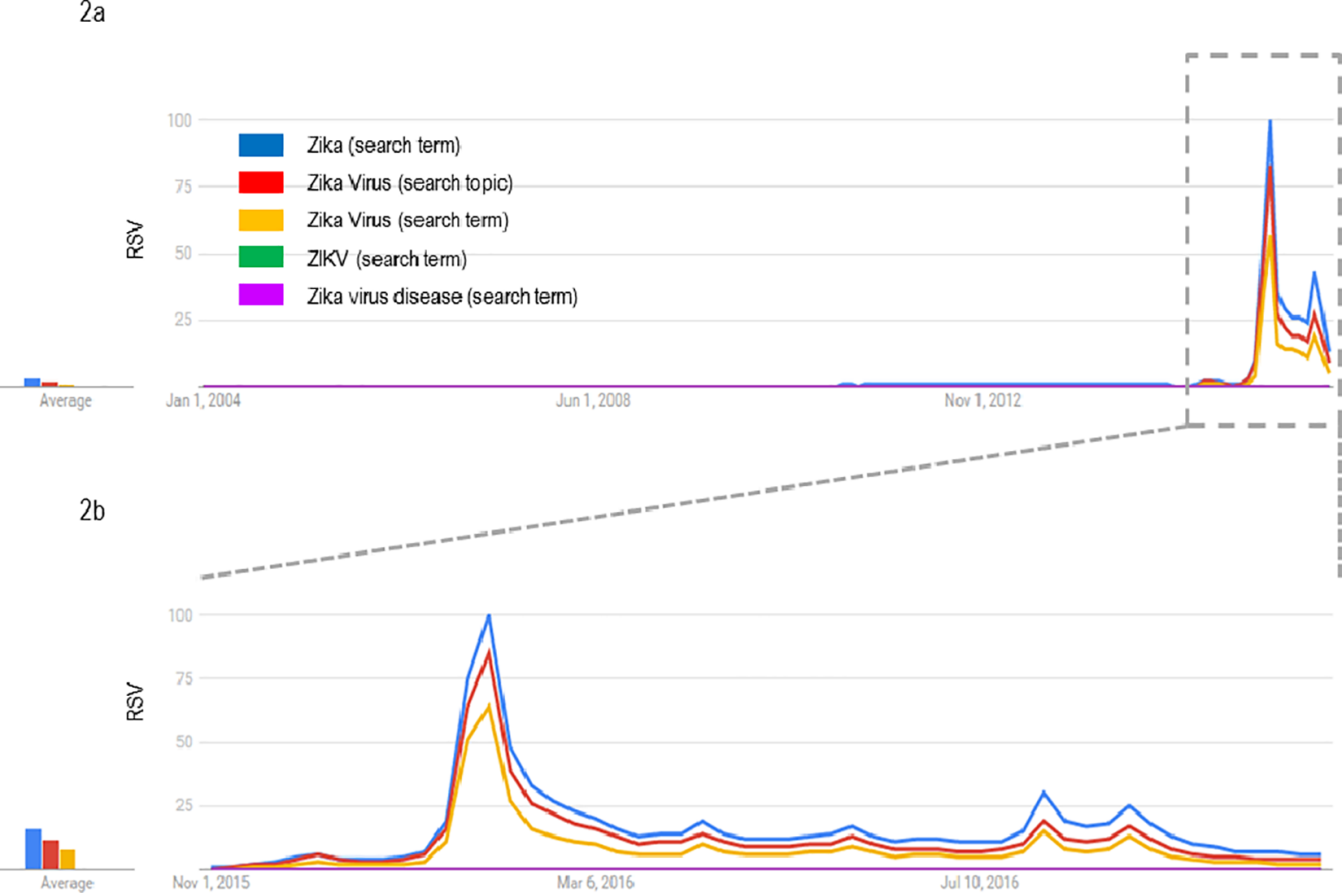
**Google Trends curves as Relatives Search Volumes (RSVs) for “Zika” and “Zika virus” (as “search term” or “search interest”) from January 2004 to June 2016 (2a) and from November 2015 to October 2016 (2b)**.

Since 2004, three search terms (namely, “Zika” as search term, and “Zika Virus” as both search term and search topic) yielded overlapping volume and temporal flux with a spike of research since the late 2015 (Fig 2A), meanwhile no queries resulted associated with “ZIKV” as search term and “Zika Virus disease” as search term. In particular, the keyword that yielded the greatest RSV was “Zika”, searched using the “search term” option.

The main spike of web searches has been registered in the period between 24^th^ January and 13^th^ February 2016 with the highest volume flux observed on 3^rd^ February 2016 (Fig 2B). This peak was preceded by a smaller peak reached in 28^th^ January 2016. Four other little increase in the volume of researches have been registered in April, June, July and August 2016. The same trend have been registered also for “Zika Virus” as search topic and search term even though with lower volume fluxes.

During the epidemic period, the GT-RSV increased markedly with a median value of 23 in comparison with a median value of 0 detected before the epidemic breakout.

**Fig 3:**
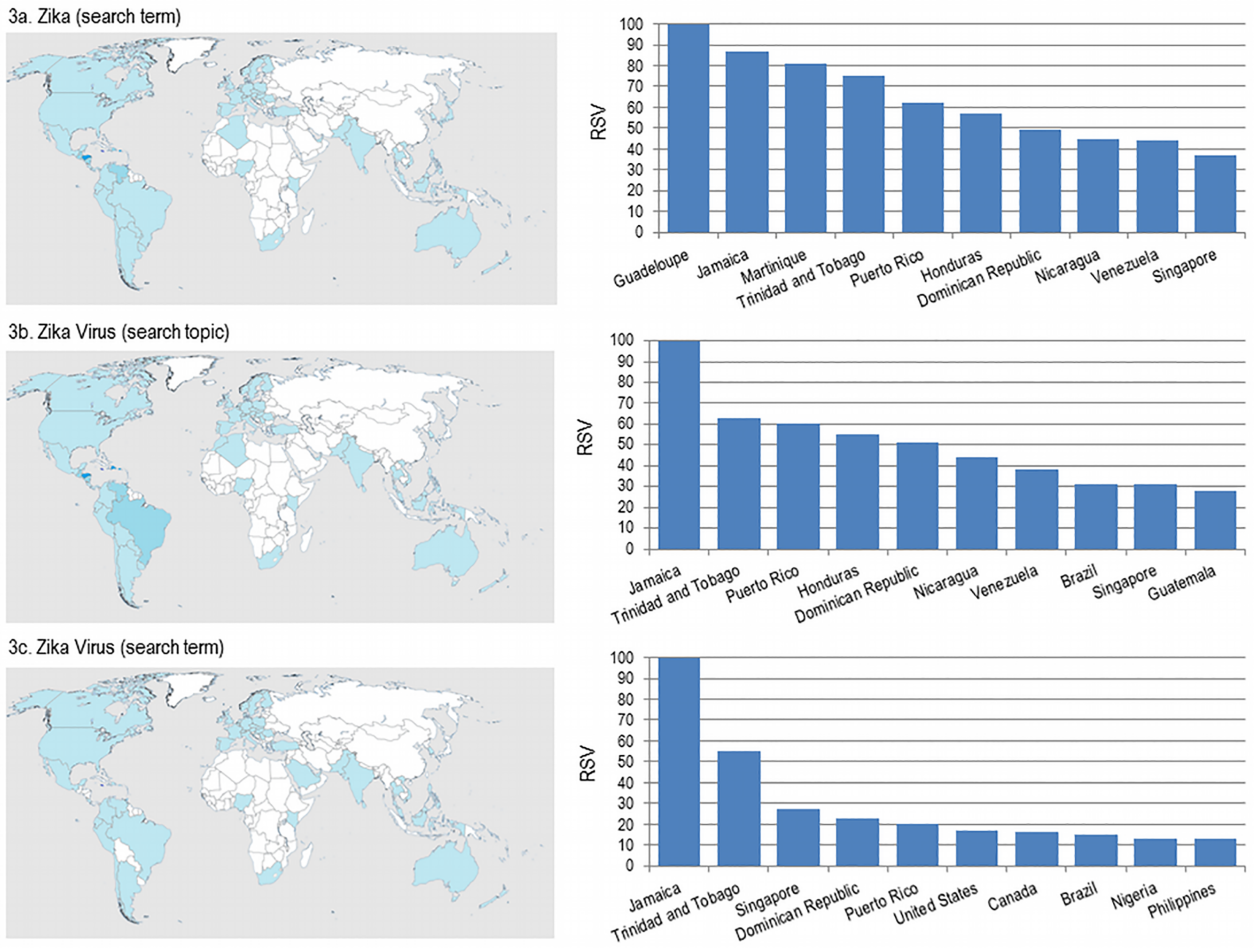
**Global interest for Zika (3a) and Zika virus-related search terms (3b and 3c): internet activities from November 2015 to October 2016 according to country** (the map was freely accessible and modifiable at the link https://commons.wikimedia.org/wiki/File:Carte_du_monde_vierge_(Allemagnes_s%C3%A9par%C3%A9es).svg).

The countries in which web searches are particularly concentrated are shown in Fig 3 and are mainly from Central and South Americas and Singapore, whilst low level of research was registered in North America, Europe, South-East Asian countries, Australia and Pacific islands.

Table 2 shows the top and rising queries for the three search terms options that yielded results. The majority of queries concerned the symptoms of the Zika virus, its vector of transmission, and its possible effect to babies, including microcephaly. Moreover, some searches were related to the diffusion of Zika virus in specific regions or countries, such as Singapore, Mexico, Brazil and Florida.

**Table 2:** Zika and Zika virus related Google Trends search queries at global level, November 2015 –October 2016.

Search option	Top queries	RSV	Rising queries	% increase
Zika (search term)	Virus	100	zika babies	Breakout
	virus zika	100	zika virus babies	Breakout
	sintomas zika	15	zika microcefalia	Breakout
	zika symptoms	10	microcefalia	Breakout
	Symptoms	10	microcephaly	Breakout
	Dengue	10	zika microcephaly	Breakout
	dengue zika	10	zika countries	Breakout
	el zika	10	zika in florida	Breakout
	the zika virus	5	zika virus microcefalia	Breakout
	zika virus symptoms	5	zika virus countries	Breakout
Zika Virus (search topic)	Zika	100	sintomas da zika	Breakout
	Virus	70	sintomas dengue	Breakout
	virus zika	70	zika virus map	Breakout
	Sintomas	10	baby zika virus	Breakout
	zika sintomas	10	chicungunha	Breakout
	zika symptoms	5	microcefalia	Breakout
	Symptoms	5	sintomas da dengue	Breakout
	Dengue	5	microcephaly	Breakout
	dengue zika	5	zika virus mexico	Breakout
	the zika virus	5	zika brasil	Breakout
Zika Virus (search term)	the zika virus	100	zika virus babies	Breakout
	zika symptoms	95	zika virus baby	Breakout
	zika virus symptoms	95	zika baby	Breakout
	Symptoms	90	microcephaly	Breakout
	virus de zika	55	zika virus microcephaly	Breakout
	zika virus sintomas	50	microcefalia	Breakout
	zika sintomas	50	zika virus countries	Breakout
	what is zika	40	virus zika singapore	Breakout
	zika virus map	35	virus singapore	Breakout
	zika babies	30	zika in florida	Breakout

RSV: Relative Search Volume

Breakout” means that the search term grew by more than 5000%.

With respect to countries searches for Zika, in S1 Table we reported the weekly RSV in the period 1^st^ November 2015 - 31^st^ October 2016 for all countries listed in the methods section. Overall, 59/80 (73.8%) countries reached the highest peak of web searches in the period between 24^th^ January and 13^th^ February 2016. Six countries reach the RSV peak in the two weeks before or after this time window.

Interestingly, in the countries where the onset of mosquito-borne Zika virus transmission was reported in early or late 2015, the majority of weekly RSV >25 were concentrated in the period between November 2015 and March 2016. In the countries where the cases were reported between January and March 2016, weekly RSV >25 were first observed since January 2016 and in the majority of countries were no longer recorded after June 2016. Of note, in some Caribbean countries, high RSV (>50) were registered in the period July-October 2016, with two countries reaching the peak of RSV in August and October, respectively. A similar pattern was observed also in the Caribbean countries that reported the first cases during the period April-June 2016. Finally, in the countries that lastly registered mosquito-borne Zika virus transmission, the majority of RSV > 25 were concentrated in the period August-October 2016, with 5 countries reaching the peak during this period.

### Twitter

Overall 3,864,395 tweets were retrieved during the study period. Almost all tweets (99.9%) were generated in the epidemic period (from November 2015 to October 2016), with most of them (53.6%) concentrating in the months of January and February 2016. With respect to the pre-epidemic period, the average monthly number of tweets increased of almost 1 million times. In Fig 1, we reported normalized value of monthly tweets.

### Wikitrends

Overall 284,903 accesses to Wikipedia page dedicated to the Zika virus were performed in the study period. The page written in English was the most consulted (98.8%). As already reported, an increasing trend could be noticed since November 2015. Approximately 68% of accesses were performed in the period November 2015 –January 2016, with a peak observable in the month of January 2016, when approximately half of the accesses (48.8%) concentrated. Noteworthy, a small peak could be detected in April 2011.

With respect to the pre-epidemic period, the average monthly number of accesses to Wikipedia increased of almost 7000%. In Fig 1, we reported normalized value of monthly accesses to Wikipedia.

### YouTube

A peak of web-interactions trough this platform could be detected in February 2016, with a small peak in August 2016. During the epidemic period, the YouTube-RSV increased markedly with a median value of 18 in comparison with a median value of 3 detected before the epidemic breakout.

In Fig 1, we reported normalized value of monthly accesses to YouTube.

### Google News

A first peak of web-searches could be detected in February 2016, with a second peak observable in August 2016. During the epidemic period, the Google News-RSV increased markedly with a median value of 54 in comparison with a median value of 0 detected before the epidemic breakout.

In Fig 1, we reported normalized value of monthly accesses to Google News.

### Correlation between all novel data streams

In Table 3, the correlations between all novel data streams are shown. All correlations resulted statistically significant, apart from the correlation between Twitter and YouTube and between Wikitrends and YouTube. Scatter plots of each correlation were presented as supplementary material (S1 File).

**Table 3:** Spearman’s correlation between all novel data streams.

		Google News	Google Trends	Twitter	Wikitrends
**Google Trends**	Correlation coefficient Significance Level P	0.897 <0.0001			
**Twitter**	Correlation coefficient Significance Level P	0.620 <0.0001	0.659 <0.0001		
**Wikitrends**	Correlation coefficient Significance Level P	0.382 0.0001	0.482 <0.0001	0.792 <0.0001	
**YouTube**	Correlation coefficient Significance Level P	0.433 <0.0001	0.302 0.0017	0.014 0.8901	-0.150 0.1415

### Correlations with real-world epidemiological data

No statistically significant correlations were found between novel data streams and global real-world epidemiological data. Both at monthly and weekly basis, the correlation between web-searches and confirmed Zika cases was negative, even though not statistically significant. At country level, a correlation between the digital interest towards the Zika virus and Zika incidence rate or microcephaly cases could be detected (raw data were available as S2 Table) (Table 4). In particular, the correlation between confirmed autochthonous cases and GT-based RSVs resulted 0.55 (p<0.0001), whilst the correlation between microcephaly cases and RSVs yielded a coefficient of 0.45 (p<0.001). Correlations between imported or suspected autochthonous cases and GT-based RSVs were not statistically significant. Correlation between Zika incidence rate (per 100,000 inhabitants) and GT-based RSV resulted negative, even though statistically borderline. Scatter plots of each correlation were presented as supplementary material (S1 File).

Concerning the generalized multivariate regression model, no statistically significant findings could be obtained. The model indicated that the number of searches was associated with HDI, however this did not reach statistical significance.

**Table 4:** Spearman’s correlation between Google Trends-generated Relative Search Volumes (RSVs) and “real-world” epidemiological data, at country level.

Epidemiological variable	RSV (p-value)
Confirmed autochthonous cases	0.546 (<0.0001)
Imported cases	0.149 (0.2908)
Incidence rate (per 100,000 inhabitants)	-0.261 (0.0615)
Microcephaly	0.454 (0.0007)
Suspected autochthonous cases	0.254 (0.0694)

## Discussion

This study analyzed the Zika-related digital web searching behavior and the social media interactions throughout the epidemic spreading, since its beginning in Brazil in the early 2015.

Indeed, novel data streams are a valuable tool to study health seeking behavior at population level, in particular as a reaction to emerging health threats [15,17].

Our research revealed that the explosive, unexpected spread of the Zika virus and the associated surge of Guillain-Barré syndrome and microcephaly cases have raised public awareness and concerns for the new aggressively emerging threat, generating since the late 2015 an extraordinary increase of related internet searches and social media interactions worldwide [29].

Specifically, the two main peaks of both searches and social media interactions, observed at global level, seemed to coincide with the news that the World Health Organization (WHO) convened at an emergency committee to discuss the “explosive” spread of the Zika virus (28^th^ January 2016), following three day after by the declaration of Zika virus as Public Health Emergency of International Concern (PHEIC) (1^st^ February 2016), and then by the notice of the first US autochthon transmission by sexual contact (3^rd^ February 2016).

A lower peak of web searches and social interactions, particularly evident by Google News, was registered in August 2016, possibly coinciding with the news related to the Brazilian Olympic Games and the spread of the epidemics in Florida.

Interestingly, the pattern of web searches observed in all the 80 countries interested by mosquito-borne or person-to-person transmission of Zika virus, with the exception of few countries and territories located in the Caribbean Region and Western Pacific Region, reached the peak between 28^th^ January and 3^rd^ February 2016. This finding seems to confirm that web search interest in Zika virus, both at the global and national levels, was strongly influenced by the declaration of a possible global health threat and the media coverage of Western Countries, as already observed for the 2014 Ebola outbreak [12].

As further proof of this, no web search activity was detectable at global level in correspondence with the local news about the first phases of the outbreak, such as the news about the first Brazilian suspected cases (April 2015) and the official declaration of the Brazilian Minister of Health which confirmed autochthonous transmission of the virus in the north-eastern part of the country (May 2015). Meanwhile, a very low volume of web researches was recorded in correspondence with the epidemiological alert issued on the 1^st^ December 2015 by the Pan American Health Organization (PAHO), urging Latin American countries to take adequate measures to prevent the virus entry, tracking its spread and actively monitoring the disease [30]. In the last two months of 2015, some spikes of web searches were detectable in few countries in Central and South America, particularly those where the onset of mosquito-borne Zika virus transmission was reported in early or late 2015. Interestingly, the propensity to search for Zika, assessed through the distribution of GT-RSV >25 for the search term “Zika”, seemed to show an “invasion dynamic” that followed the geographical spread of Zika virus across the Americas and the other affected countries. As reported, the earlier searches were observed in Brazil, where the current pandemic originated, and then they were documented in the other countries interested by the pandemic with a temporal pattern similar to that of the first reported Zika cases by countries. In fact, in the countries where the first Zika cases were reported in 2016, RSV >25 were not documented before January 2016, and some countries reached the highest peak of searches between March and October 2016, possibly related to local events or news.

It is worth noting that queries were geospatially concentrated in the South and Central America, whilst little trace of web-related activities is detectable in North American, African, European or Asian and Pacific countries. Although the web-related activities were concentrated in the area most affected with the spread of Zika virus, a correlation between the number of Zika virus cases or microcephaly cases and the volume of web researches at national level has not been demonstrated. Nevertheless, it has been proposed that the volume of researches about global health threat may depend not exclusively on the epidemiological diffusion of the disease but also on its perceived severity and the internet as well as other mass media penetration at country level.

Interestingly, the Zika virus-associated searches concerned three main topics, namely the symptoms of the disease caused by this organism, the vector of the disease and other *Aedes*-transmitted diseases such as Dengue and Chikungunya, and the effect of the Zika virus disease on the newborn, particularly the microcephaly which was recently associated with the virus. This latter finding is consistent with the results of Khatua and Khatua, that performed volumetric and text mining analyses on a corpus of 975,752 tweets posted between 25^th^ January and 14^th^ February 2016, finding that most tweets concern serious long-term effects of the Zika virus, such as microcephaly in newborns, and topics such as pregnancy and abortion [26]. Fu and colleagues reported the same conclusions in their computational content analysis of ZIKV-related English Tweets in late January 2016 [25].

These web queries, if on one hand underline the need of the public opinion to know and remain updated on this emerging pathogen, on the other hand make evident some information gaps. These queries seem to be most exclusively reactive and not proactive, which would the case if users searched, for example, how to prevent this infection and which precautions to adopt, like insect repellent, clothing, screens on windows and mosquito nets on beds, as well as eliminating/avoiding any receptacle site that can act as breeding site such as surfaces or grounds with standing water [31].

The reactive nature of digital activities towards the Zika virus seems to be confirmed by a number of considerations. First, all novel data streams showed highly statistically significant correlation among each other and captured a similar, consistent web-related behavior. On one hand, such overlap could be expected in that, for example, the Wikipedia page is generally the first or one of the first returned results from a Google query. On the other hand, these novel data streams capture the behavior of different population segments (lay people, scientific community, etc.) and the degree of overlap depends on the particular search-term. Segev and Sharon have shown that basically search terms can be categorized in “*ad hoc*” and in cyclic terms [32]. An outbreak represents an *ad hoc* term and, in fact, we observed a large overlap. Second, we found not-statistically significant correlations between novel data stream signals with real-world epidemiological data at global level. At country level, we detected a statistically significant association between Internet searches and absolute number of Zika cases and microchephaly, but not between health-information seeking behavior and Zika incidence. The association within number of searches and HDI, suggested that most searches were performed in rich and developed countries, which were less involved by the Zika epidemic outbreak. These findings seem to confirm that the link between the epidemiology of public health problems and the volumes of internet searches and social media interactions is complex. Recently, Althouse and colleagues illustrated that the relationship between novel data stream signals and disease incidence is shaped by several factors including user behavior (i.e. propensity to search, what terms are chosen to search etc.), user demographics, external forces on user behavior (changing disease severity or its perception, changing press coverage, availability of internet, mass media penetration, etc.) and public health interventions [17].

Remarkably, global and local health agencies, as well as by public health practitioners, could practically leverage web searches and social media interactions to investigate the disease-related information needs, social and behavioral barriers to infection control, and misinformation. Sharma and collaborators carried out a research on Facebook posts related to the Zika virus and found that the most popular posts contained misleading and inaccurate information [33]. Moreover, these tools could be used to analyze the sentiment and the risk perception associated with health-threatening events such as Zika pandemic outbreak [12].

Furthermore, public health agencies could exploit these tools to spread healthcare-related information quickly, effectively and cheaply, both in developed and developing countries, where the diffusion of new technologies and social networks among the population is rapidly increasing. Google Trends as well as Twitter could be used as a proxy of the proper diffusion of strategies based on health education messages and to determine the level of uptake of information about a public health event [15,17].

This research has a number of limitations. First, the precise algorithm used by GT, Google News and YouTube is not publicly known and this hinders further processing and manipulation of the data, as already reported. Further, these data are provided as relative, normalized figures, and not as absolute, crude data. Because of this normalization procedure, the research strictly depends on the chosen time window and geographic location. On the other hand, in order to overcome this shortcoming, we compared GT, Google News and YouTube search volumes with the pattern captured by other social media and we found that this behavior is consistent and reproducible among different web platforms. Moreover and as already highlighted, since epidemiological data are uncertain, the performed correlations should be interpreted with caution.

In conclusion, GT as well as the other novel data streams documented a consistent, increasing public interest and reaction to the current Zika virus outbreak. The public opinion is following with great concern what is happening and seems to be worried by the alert of teratogenicity of the Zika virus. GT and the other Internet tools could be helpful for stakeholders and health authorities in collecting the concerns of public opinion and reply to them, disseminating key information [34,35].

## Supporting information

S1 Table Weekly Relative Search Volumes (RSV) from Google Trends in the period 1^st^ November 2015 - 31^st^ October 2016 for all countries. (i) Countries where autochthonous mosquito-borne Zika virus infections have been reported, (ii) countries with possible endemic transmission or evidence of local mosquito-borne Zika infections in 2016, (iii) countries with evidence of local mosquito-borne Zika infections in or before 2015, but without documentation of cases in 2016, or outbreak terminated, (iv) countries with evidence of person-to-person transmission of Zika virus, other than mosquito-borne transmission.
(PDF)

S2 Table Raw data about Relative Search Volumes (RSV) from Google Trends in the period 1^st^ November 2015 - 31^st^ October 2016, cases of microcephaly, suspected autochthonous cases, confirmed autochthonous cases, incidence rate, imported cases, number of inhabitants and Human Development Index, for each country belonging to Pan American Health Organization (PAHO), used for multivariable regression models. (PDF)

S1 File Scatter plots of correlations between all novel data streams an between novel data streams and real-world epidemiological data. (PDF)

## References

[1] The Lancet. Zika virus: a new global threat for 2016. Lancet 2016;387:96 doi: 10.1016/S0140-6736(16)00014-3 10.1016/S0140-6736(16)00014-3 26841979

[2] Dick GW, Kitchen SF, Haddow AJ. Zika virus. I. Isolations and serological specificity. Trans R Soc Trop Med Hyg 1952;46:509–520. 12995440 10.1016/0035-9203(52)90042-4

[3] Duffy MR, Chen TH, Hancock WT, Powers AM, Kool JL, Lanciotti RS, et al Zika virus outbreak on Yap Island, Federated States of Micronesia. N Engl J Med 2009;360:2536–43. doi: 10.1056/NEJMoa0805715 19516034 10.1056/NEJMoa0805715

[4] Cao-Lormeau VM, Roche C, Teissier A, Robin E, Berry AL, Mallet HP, et al Zika virus, French polynesia, South pacific, 2013. Emerg Infect Dis 2014;20:1085–6. doi: 10.3201/eid2006.140138 24856001 10.3201/eid2006.140138 PMC4036769

[5] World Health Organization (WHO). Zika virus, microcephaly and Guillain-Barré syndrome. Situation Report: 3 November 2016. Available from: http://apps.who.int/iris/bitstream/10665/250724/1/zikasitrep3Nov16-eng.pdf?ua=1 Cited 26 Jan 2017.

[6] World Health Organization (WHO). Zika virus, microcephaly and Guillain-Barré syndrome. Situation report: 2 June 2016. Available from: http://apps.who.int/iris/bitstream/10665/208816/1/zikasitrep_2Jun2016_eng.pdf?ua=1 Cited 26 Jan 2017.

[7] Waddell LA, Greig JD. Scoping Review of the Zika Virus Literature. PLoS One 2016; 11(5):e0156376 doi: 10.1371/journal.pone.0156376 27244249 10.1371/journal.pone.0156376 PMC4887023

[8] Fleming-Dutra KE, Nelson JM, Fischer M, Staples JE, Karwowski MP, Mead P, et al Update: Interim Guidelines for Health Care Providers Caring for Infants and Children with Possible Zika Virus Infection—United States, February 2016. MMWR Morb Mortal Wkly Rep 2016;65:182–7. doi: 10.15585/mmwr.mm6507e1 26914500 10.15585/mmwr.mm6507e1

[9] Cao-Lormeau VM, Blake A, Mons S, Lastère S, Roche C, Vanhomwegen J, et al Guillain-Barré Syndrome outbreak associated with Zika virus infection in French Polynesia: a case-control study. Lancet 2016;387:1531–9. doi: 10.1016/S0140-6736(16)00562-6 26948433 10.1016/S0140-6736(16)00562-6 PMC5444521

[10] Rasmussen SA, Jamieson DJ, Honein MA, Petersen LR. Zika Virus and Birth Defects—Reviewing the Evidence for Causality. N Engl J Med 2016;374:1981–7. doi: 10.1056/NEJMsr1604338 27074377 10.1056/NEJMsr1604338

[11] Johansson MA, Mier-y-Teran-Romero L, Reefhuis J, Gilboa SM, Hills SL. Zika and the Risk of Microcephaly. N Engl J Med 2016;375:1–4. doi: 10.1056/NEJMp1605367 27222919 10.1056/NEJMp1605367 PMC4945401

[12] Alicino C, Bragazzi NL, Faccio V, Amicizia D, Panatto D, Gasparini R, et al Assessing Ebola-related web search behaviour: insights and implications from an analytical study of Google Trends-based query volumes. Infect Dis Poverty. 2015 12 10;4:54 doi: 10.1186/s40249-015-0090-9 26654247 10.1186/s40249-015-0090-9 PMC4674955

[13] Wong R, Harris JK, Staub M, Bernhardt JM. Local Health Departments Tweeting About Ebola: Characteristics and Messaging. J Public Health Manag Pract. 2017;23:e16–e24. doi: 10.1097/PHH.0000000000000342 26334537 10.1097/PHH.0000000000000342

[14] Towers S, Afzal S, Bernal G, Bliss N, Brown S, Espinoza B, et al Mass Media and the Contagion of Fear: The Case of Ebola in America. PLoS One 2015;10:e0129179 doi: 10.1371/journal.pone.0129179 26067433 10.1371/journal.pone.0129179 PMC4465830

[15] Ayers JW, Althouse BM, Dredze M. Could Behavioral Medicine Lead the Web Data Revolution? JAMA. 2014;311:1399–1400. doi: 10.1001/jama.2014.1505 24577162 10.1001/jama.2014.1505 PMC4670613

[16] Santillana M, Zhang DW, Althouse BM, Ayers JW. What can digital disease detection learn from (an external revision to) Google Flu Trends? Am J Prev Med 2014;47:341–7. doi: 10.1016/j.amepre.2014.05.020 24997572 10.1016/j.amepre.2014.05.020

[17] Althouse BM, Scarpino SV, Meyers LA, Ayers JW, Bargsten M, Baumbach J, et al Enhancing disease surveillance with novel data streams: challenges and opportunities. EPJ Data Science. 2015;4:1 doi: 10.1140/epjds/s13688-015-0054-0 27990325 10.1140/epjds/s13688-015-0054-0 PMC5156315

[18] Ayers JW, Westmaas JL, Leas EC, Benton A, Chen Y, Dredze M, et al Leveraging Big Data to Improve Health Awareness Campaigns: A Novel Evaluation of the Great American Smokeout. JMIR Public Health Surveill 2016;2:e16 doi: 10.2196/publichealth.5304 27227151 10.2196/publichealth.5304 PMC4869240

[19] Brownstein JS, Freifeld CC, Madoff LC. Digital disease detection—harnessing the Web for public health surveillance. N Engl J Med 2009;360:2153–5, 2157. doi: 10.1056/NEJMp0900702 19423867 10.1056/NEJMp0900702 PMC2917042

[20] Chew C, Eysenbach G. Pandemics in the age of Twitter: content analysis of Tweets during the 2009 H1N1 outbreak. PLoS One. 2010;5:e14118 doi: 10.1371/journal.pone.0014118 21124761 10.1371/journal.pone.0014118 PMC2993925

[21] Olson DR, Konty KJ, Paladini M, Viboud C, Simonsen L. Reassessing Google Flu Trends data for detection of seasonal and pandemic influenza: a comparative epidemiological study at three geographic scales. PLoS Comput Biol. 2013;9(10):e1003256 doi: 10.1371/journal.pcbi.1003256 24146603 10.1371/journal.pcbi.1003256 PMC3798275

[22] Nuti SV, Wayda B, Ranasinghe I, Wang S, Dreyer RP, Chen SI, et al The use of google trends in health care research: a systematic review. PLoS One 2014;9(10):e109583 doi: 10.1371/journal.pone.0109583 25337815 10.1371/journal.pone.0109583 PMC4215636

[23] Hossain L, Kam D, Kong F, Wigand RT, Bossomaier T. Social media in Ebola outbreak. Epidemiol Infect 2016;144:2136–43. doi: 10.1017/S095026881600039X 26939535 10.1017/S095026881600039X PMC9150578

[24] Southwell BG, Dolina S, Jimenez-Magdaleno K, Squiers LB, Kelly BJ. Zika Virus-Related News Coverage and Online Behavior, United States, Guatemala, and Brazil. Emerg Infect Dis 2016;22:1320–1. doi: 10.3201/eid2207.160415 27100826 10.3201/eid2207.160415 PMC4918164

[25] Fu KW, Liang H, Saroha N, Tse ZT, Ip P, Fung IC. How people react to Zika virus outbreaks on Twitter? A computational content analysis. Am J Infect Control 2016;44(12):1700–1702. doi: 10.1016/j.ajic.2016.04.253 27566874 10.1016/j.ajic.2016.04.253

[26] Khatua A, Khatua A. Immediate and Long-term Effects of 2016 Zika Outbreak: A Twitter-based Study. 2016 IEEE 18th International Conference on e-Health Networking, Applications and Services (Healthcom) 2016;1–6. 10.1109/HealthCom.2016.7749496.

[27] Smallwood C. The Complete Guide to Using Google in Libraries: Instruction, Administration and Staff Productivity. 1st ed. Rowman & Littlefield Lanham, Maryland, USA 2015.

[28] Pan American Health Organization (PAHO). Zika cumulative cases at 3 november 2016. Available from: http://www.paho.org/hq/index.php?option=com_docman&task=doc_view&Itemid=270&gid=36752&lang=en Cited 26 January 2017.

[29] Majumder MS, Santillana M, Mekaru SR, McGinnis DP, Khan K, Brownstein JS. Utilizing Nontraditional Data Sources for Near Real-Time Estimation of Transmission Dynamics During the 2015–2016 Colombian Zika Virus Disease Outbreak. JMIR Public Health Surveill 2016;2:e30 doi: 10.2196/publichealth.5814 27251981 10.2196/publichealth.5814 PMC4909981

[30] Valero N. Zika virus: Another emerging arbovirus in Venezuela? Invest Clin 2015;56:241–2. 26710538

[31] Brown C. Zika virus outbreaks in Asia and South America. CMAJ 2016;188(2):E34 doi: 10.1503/cmaj.109-5212 26696621 10.1503/cmaj.109-5212 PMC4732977

[32] Segev E, Sharon AJ. Temporal patterns of scientific information-seeking on Google and Wikipedia. Public Underst Sci. 2016;pii:0963662516648565. 10.1177/0963662516648565 27208006

[33] Sharma M, Yadav K, Yadav N, Ferdinand KC. Zika virus pandemic-analysis of Facebook as a social media health information platform. Am J Infect Control. 2016;pii: S0196-6553(16)30918-X. doi: 10.1016/j.ajic.2016.08.022 27776823 10.1016/j.ajic.2016.08.022

[34] Lazard AJ, Scheinfeld E, Bernhardt JM, Wilcox GB, Suran M. Detecting themes of public concern: a text mining analysis of the Centers for Disease Control and Prevention's Ebola live Twitter chat. Am J Infect Control 2015;43(10):1109–11. doi: 10.1016/j.ajic.2015.05.025 26138998 10.1016/j.ajic.2015.05.025

[35] Wojda TR, Valenza PL, Cornejo K, McGinley T, Galwankar SC, Kelkar D, et al The Ebola Outbreak of 2014–2015: From Coordinated Multilateral Action to Effective Disease Containment, Vaccine Development, and Beyond. J Glob Infect Dis 2015;7:127–38. doi: 10.4103/0974-777X.170495 26752867 10.4103/0974-777X.170495 PMC4693303

