# Supplementary material for: Global reaction to the recent outbreaks of Zika virus: Insights from a Big Data analysis
Source: PLoS One. 2017 Sep 21;12(9):e0185263. doi: 10.1371/journal.pone.0185263 (PMC5608413; doi:10.1371/journal.pone.0185263)
Supplement: S1 Table — (i) Countries where autochthonous mosquito-borne Zika virus infections have been reported, (ii) countries with possible endemic transmission or evidence of local mosquito-borne Zika infections in 2016, (iii) countries with evidence of local mosquito-borne Zika infections in or before 2015, but without documentation of cases in 2016, or outbreak terminated, (iv) countries with evidence of person-to-person transmission of Zika virus, other than mosquito-borne transmission. (PDF) [file pone.0185263.s001.pdf]

Week	Brazil	Honduras	Colombia	El Salvador	Guatemala	Mexico	Paraguay	Suriname	Venezuela	Papua New	Vanuatu	Solomon I	Cape Verde	French Gui	Martinique	Panama	Puerto Rico	Samoa	Bolivia	U.S. Virgin	Dominican	Costa Rica	Guadeloup	St. Martin	Nicaragua	Barbados
01/11/15	3	0	8	30	0	0	2	15	1	0	0	0	61	0	0	0	0	0	0	0	0	0	0	0	0	0
08/11/15	4	0	9	42	1	1	1	8	1	0	0	0	23	0	5	0	1	0	1	0	16	1	2	0	1	0
15/11/15	11	1	7	35	5	8	1	17	2	0	0	0	31	20	4	0	0	0	1	0	6	0	2	0	6	0
22/11/15	17	8	9	56	54	4	14	15	3	0	0	0	22	9	3	1	0	0	1	0	2	2	2	0	13	0
29/11/15	44	8	13	75	22	3	11	26	8	0	0	0	43	6	2	12	0	0	4	0	2	3	2	0	8	4
06/12/15	59	4	17	66	23	2	10	15	14	0	0	0	18	6	4	3	1	0	2	0	5	4	2	0	5	4
13/12/15	36	10	20	55	18	1	9	16	24	0	0	0	14	15	5	2	2	0	2	0	18	2	2	0	5	6
20/12/15	26	9	20	45	10	1	2	11	27	0	0	0	11	24	25	1	0	0	2	0	5	1	18	18	3	8
27/12/15	26	10	27	36	13	1	5	35	41	0	0	0	11	13	12	4	11	0	4	11	5	1	5	17	1	11
03/01/16	24	13	42	47	19	2	6	29	43	0	0	0	18	6	16	3	11	0	18	22	15	1	7	17	3	13
10/01/16	24	19	40	73	20	2	18	35	52	0	0	0	9	12	32	3	7	0	20	22	40	3	18	17	3	23
17/01/16	29	32	100	100	37	17	35	78	69	0	0	0	18	20	68	31	35	31	35	17	53	24	60	18	15	72
24/01/16	50	35	87	93	88	47	64	100	85	22	89	38	61	61	92	61	71	89	85	88	100	85	67	61	100	94
31/01/16	91	100	99	83	100	100	100	45	100	100	100	69	100	100	100	100	100	100	100	100	92	100	100	95	98	100
07/02/16	99	67	58	48	55	31	57	45	77	68	31	100	54	78	67	41	73	49	78	68	57	55	62	48	58	69
14/02/16	100	36	39	30	41	23	37	32	71	41	33	35	62	66	55	38	43	50	70	35	37	32	53	46	34	50
21/02/16	89	22	32	28	26	20	35	23	58	30	29	32	60	45	52	32	30	50	42	36	35	59	48	16	18	52
28/02/16	79	22	30	18	21	20	44	21	47	9	29	32	41	31	57	32	23	26	37	30	27	38	34	57	20	39
06/03/16	71	19	29	20	19	19	52	19	36	45	53	32	21	33	40	38	24	25	34	24	27	33	33	31	14	31
13/03/16	56	22	22	20	22	14	39	15	29	28	38	30	17	31	24	27	20	24	29	34	28	20	24	31	14	16
20/03/16	48	10	25	15	19	8	28	18	18	10	27	28	13	33	36	23	16	24	23	46	26	18	31	39	8	17
27/03/16	51	11	24	18	19	8	33	6	24	29	30	28	17	18	27	22	17	54	18	37	34	22	24	23	7	21
03/04/16	50	11	20	11	16	11	21	3	20	18	40	28	9	30	36	20	11	26	20	28	52	15	14	27	7	16
10/04/16	46	10	17	14	15	10	20	7	16	18	50	28	13	5	26	19	18	26	24	29	54	13	34	31	10	15
17/04/16	36	11	16	12	12	8	19	8	13	9	25	28	17	19	34	12	14	24	14	34	61	18	24	39	6	5
24/04/16	32	8	16	10	13	7	9	7	12	9	25	27	9	18	37	10	19	24	17	17	53	17	21	32	8	16
01/05/16	24	9	13	10	13	7	7	4	11	23	27	27	17	22	32	11	31	21	14	40	47	17	27	16	7	13
08/05/16	22	9	13	10	19	7	7	6	12	0	28	26	13	16	15	10	38	21	12	28	52	15	32	16	10	20
15/05/16	20	12	13	9	17	7	8	11	13	0	28	26	13	32	28	10	33	19	7	31	45	19	43	39	10	10
22/05/16	17	11	12	9	14	7	7	4	14	0	0	26	22	13	27	7	27	19	7	15	42	22	45	66	9	15
29/05/16	16	13	13	6	17	8	7	6	15	0	0	0	23	10	13	6	26	25	9	48	46	21	44	15	13	20
05/06/16	13	12	11	8	23	7	4	5	14	0	0	0	17	16	25	11	27	24	3	22	53	17	45	68	13	15
12/06/16	11	12	9	12	17	7	3	2	13	10	0	0	13	24	22	9	29	22	5	41	44	18	58	61	12	14
19/06/16	10	11	8	9	21	7	6	6	11	9	0	0	27	32	16	8	33	23	7	30	43	16	53	17	10	9
26/06/16	9	12	8	5	25	8	2	4	11	9	25	0	23	5	15	7	53	23	6	41	34	17	63	47	14	17
03/07/16	9	10	9	10	34	8	6	7	9	9	26	0	19	16	9	7	50	26	5	51	33	11	49	34	16	11
10/07/16	7	12	6	8	30	8	1	4	8	9	26	0	18	6	21	4	42	0	5	49	22	14	38	25	30	9
17/07/16	8	12	5	8	31	8	2	4	7	9	26	0	14	9	11	5	51	0	6	46	16	18	47	51	33	7
24/07/16	7	15	5	5	33	10	7	6	6	9	26	0	24	19	18	6	46	0	5	76	17	34	46	58	40	12
31/07/16	9	13	4	6	29	12	5	6	6	9	26	48	19	9	20	5	57	0	4	81	15	19	45	44	33	16
07/08/16	12	12	6	9	33	14	4	4	6	10	26	0	14	19	20	3	55	0	2	54	11	17	45	52	32	20
14/08/16	8	11	4	10	38	18	2	4	5	10	26	0	15	13	10	4	52	0	5	37	10	24	36	70	27	21
21/08/16	7	10	4	7	33	21	2	4	4	11	26	0	10	6	9	5	46	0	3	52	12	17	26	60	24	20
28/08/16	6	8	3	5	28	22	4	3	4	10	26	0	14	5	6	3	36	0	3	52	7	15	19	100	16	30
04/09/16	6	7	3	2	24	20	1	2	3	10	23	0	38	6	9	3	32	0	3	39	9	14	13	38	16	13
11/09/16	6	6	3	4	24	19	2	2	3	10	24	0	32	6	8	2	29	0	3	44	9	13	16	36	12	32
18/09/16	6	5	3	5	19	16	1	2	2	11	25	0	67	5	7	2	26	0	4	37	6	13	16	39	10	20
25/09/16	5	4	2	6	19	17	1	2	2	10	26	0	14	11	7	3	22	0	4	20	5	13	9	63	6	24
02/10/16	5	4	2	7	12	20	3	2	2	10	28	0	9	5	10	3	22	0	4	50	3	10	7	26	5	34
09/10/16	5	5	2	3	12	19	4	3	2	10	0	0	22	5	9	5	20	0	5	55	5	11	10	27	7	20
16/10/16	6	4	3	6	15	19	2	4	3	10	0	29	13	5	14	3	19	0	4	49	5	9	15	37	4	25
23/10/16	6	8	2	4	12	18	4	2	3	10	0	28	9	5	5	3	16	0	4	58	5	9	4	17	3	21
30/10/16	7	4	2	3	8	15	2	2	3	0	0	0	9	8	3	1	15	0	7	42	5	7	11	30	5	19

Legend

Countries with authocthonous confirmed vector borne transmission of Zika virus prior October 2015
Countries with authocthonous confirmed vector borne transmission of Zika virus in October-December 2015
Countries with authocthonous confirmed vector borne transmission of Zika virus in January-March 2016
Countries with authocthonous confirmed vector borne transmission of Zika virus in April-June 2016
Countries with authocthonous confirmed vector borne transmission of Zika virus in July-October 2016

Countries reporting person-to-person Zika virus transmission since February 2016
Countries with possible endemic transmission or evidence of local mosquito-borne Zika infections in 2016
Countries with evidence of local mosquitoborne Zika infections in or before 2015, but without documentation of cases in 2016, or outbreak terminated

Relative Search Volume (RSV) between 26 and 50
Relative Search Volume (RSV) between 51 and 75
Relative Search Volume (RSV) between 76 and 100

Reference

World Health Organization (WHO). Zika virus, microcephaly and Guillain-Barré syndrome. Situation Report: 3 November 2016. Available at http://apps.who.int/iris/bitstream/10665/250724/1/zikasitrep3Nov16-eng.pdf?ua=1 (accessed 2 Dec 2016)

Maldives	Ecuador	Guyana	Jamaica	Curacao	American S	Haiti	Tonga	Peru	Marshall Is	Micronesia	St. Vincent	Sint Maarte	Trinidad &	Aruba	Cuba	Fiji	St. Barthele	Belize	St. Lucia	Argentina	Grenada	Guinea-Bissau	Anguilla	Bahamas	Cayman Isl	United Stat	Turks & Cai	Antigua &
0	1	0	0	5	0	0	0	0	0	0	0	0	0	0	0	0	0	0	0	0	0	0	0	0	0	0	0	0
0	2	0	3	4	0	4	0	0	0	0	0	0	5	10	3	0	0	0	8	0	5	0	0	0	0	0	0	0
0	1	0	3	4	0	2	0	0	0	0	0	0	1	15	3	0	0	5	10	0	8	0	0	0	0	0	0	0
0	1	0	1	9	0	2	0	0	0	0	0	0	1	19	4	0	0	5	12	0	5	0	0	0	0	0	0	0
0	1	4	1	5	0	2	0	1	0	0	7	0	2	17	5	0	0	5	14	2	5	0	0	0	0	0	0	8
0	1	7	4	5	0	4	0	2	0	0	7	0	2	17	5	0	0	0	16	6	17	0	0	4	0	0	0	12
0	2	10	5	5	0	11	0	1	0	0	7	0	2	17	12	0	0	0	18	4	6	0	0	6	7	0	0	8
0	2	13	2	8	0	12	0	1	0	0	8	0	1	23	11	0	38	0	21	1	6	0	0	7	7	1	0	8
0	1	13	4	10	0	20	0	1	0	0	14	0	5	27	11	0	15	0	25	2	6	0	0	9	7	1	0	8
0	1	13	4	5	0	78	0	1	0	0	13	0	4	32	5	0	19	0	8	2	7	0	0	11	6	1	0	8
0	19	47	12	13	0	53	0	4	0	0	13	21	4	17	6	0	18	4	16	3	8	0	27	4	12	4	12	20
34	27	52	69	100	0	58	14	15	0	0	30	57	15	45	24	5	53	11	55	19	35	0	18	9	25	16	17	52
90	53	99	70	80	55	74	29	100	0	0	59	100	92	68	55	55	94	61	100	97	44	0	100	65	70	71	49	100
100	100	100	100	98	26	100	100	85	46	66	82	97	100	100	100	100	100	100	95	100	86	47	60	51	66	100	52	60
33	56	51	38	73	57	60	81	41	30	87	36	65	39	91	56	42	58	36	37	42	36	100	62	34	9	49	34	53
21	51	98	31	37	100	38	18	20	30	84	32	31	51	69	41	62	53	20	38	18	31	45	15	19	21	28	10	51
11	30	44	18	30	100	36	17	13	100	93	46	38	33	30	63	14	42	22	34	15	15	45	28	24	12	24	33	35
8	22	50	15	32	20	28	25	9	65	51	20	10	19	76	77	15	27	16	21	27	9	0	35	8	17	19	34	33
8	17	27	20	19	21	33	12	12	33	44	11	31	18	36	45	19	17	17	15	35	16	0	15	15	6	17	11	7
5	13	36	18	21	22	16	13	12	35	58	20	26	16	58	42	29	45	7	22	38	10	0	15	15	6	13	10	18
16	9	31	15	17	22	21	16	11	37	72	15	37	18	53	14	16	31	10	19	16	5	0	15	8	6	12	22	14
10	8	26	12	16	22	18	20	11	38	86	12	10	16	17	13	42	49	16	7	16	5	0	15	13	17	12	10	7
10	7	28	13	8	22	12	13	9	39	100	18	11	20	25	10	26	17	23	62	12	5	0	16	10	12	11	11	14
5	7	20	12	15	23	14	13	8	45	40	24	28	20	27	8	46	35	25	59	10	10	0	23	17	6	26	11	21
8	6	30	18	6	48	8	0	10	52	40	18	11	14	35	6	32	34	19	33	7	5	0	26	7	18	16	12	13
10	4	22	9	7	19	12	0	7	46	40	19	12	11	25	11	30	12	17	27	6	22	57	29	12	12	14	19	17
5	5	9	13	11	19	10	0	7	46	40	16	11	8	38	12	13	36	13	26	5	38	47	32	17	6	15	15	50
5	5	13	16	14	19	9	0	7	46	0	15	21	12	25	8	18	29	6	22	4	15	47	16	10	12	17	21	43
16	5	15	22	14	21	8	0	6	42	0	24	37	8	18	8	22	12	26	56	4	25	47	21	19	22	19	12	29
5	4	13	23	8	28	12	0	6	42	0	30	41	8	17	6	31	54	20	59	3	23	47	25	10	18	21	28	17
11	5	31	54	22	35	9	0	5	42	0	17	21	10	17	13	23	24	26	45	3	17	47	17	13	11	30	27	24
17	6	10	79	11	43	10	0	6	0	0	27	20	12	12	12	15	30	27	44	3	14	47	39	12	21	22	44	14
5	5	23	68	25	45	13	0	7	0	0	40	20	13	17	10	13	34	26	51	2	26	47	61	15	18	17	28	14
10	4	18	69	6	48	7	0	7	0	0	50	17	20	9	14	10	39	27	86	2	44	48	31	17	21	19	11	23
10	6	16	55	13	35	11	0	5	0	50	39	23	21	11	6	20	38	16	90	2	70	48	18	11	21	20	11	32
5	5	4	38	11	24	9	0	4	0	0	47	11	31	44	4	18	25	29	60	2	100	44	18	9	31	17	12	44
5	6	18	43	15	22	6	11	4	0	0	80	17	42	20	3	11	13	18	76	1	83	44	65	9	18	17	12	34
5	5	16	38	8	22	10	0	4	0	0	69	45	61	20	5	9	50	16	52	1	67	48	17	17	25	18	22	42
5	4	5	34	9	26	6	0	3	40	0	83	22	64	45	7	13	39	14	86	2	75	52	17	33	25	24	54	34
5	6	10	42	21	0	6	0	4	69	0	82	28	89	28	6	4	27	12	89	5	64	57	33	80	42	59	100	63
5	3	9	34	8	0	8	0	4	37	42	100	27	72	48	10	16	13	19	50	3	37	0	17	69	100	36	38	50
10	6	9	24	20	0	5	0	3	37	0	46	39	71	17	3	4	36	27	48	2	44	0	16	47	49	31	46	58
7	5	7	24	20	0	7	0	3	0	0	67	22	54	45	16	11	20	25	35	2	25	0	16	100	63	36	24	46
30	5	14	19	26	0	7	0	3	0	0	25	33	45	11	8	11	12	19	42	2	17	0	26	71	53	27	24	47
13	2	7	16	14	0	7	0	2	0	0	31	39	39	17	10	18	12	31	26	2	23	0	18	73	40	24	19	52
17	3	7	14	21	23	5	0	2	0	0	17	27	29	25	14	13	11	32	11	3	22	0	18	51	19	18	13	65
20	3	7	13	10	21	3	0	1	0	0	20	41	24	17	11	4	15	21	7	2	15	0	18	47	46	13	26	59
70	3	5	11	11	21	5	11	2	0	0	11	21	17	17	16	6	19	39	11	2	14	0	18	41	17	11	27	68
16	2	3	7	14	21	3	0	2	0	0	19	15	15	12	5	6	23	31	8	2	5	0	17	18	14	10	12	62
12	2	9	10	34	21	9	0	2	0	0	15	21	13	27	9	6	12	18	37	2	20	0	17	25	12	8	12	55
9	3	4	7	29	0	6	0	2	0	0	6	27	9	26	8	13	12	23	7	2	8	0	18	22	30	8	12	25
5	3	14	7	35	0	6	0	1	0	46	6	27	7	17	14	11	13	18	8	2	7	0	17	15	24	7	12	20
16	3	6	8	51	0	4	0	2	0	0	0	10	8	14	12	11	25	8	7	2	7	0	0	16	0	7	11	33

British Virg	Singapore	St. Kitts & I	Palau	Canada	Chile	France	Germany	Italy	Netherland	Portugal	Spain	New Zealar	Indonesia	Thailand	Malaysia	New Caled	Philippines	Viet Nam	Gabon	Bangladesh	Cambodia	Cook Islanc	French Poly	Laos	Worldwide
0	0	0	0	0	0	0	0	0	0	1	0	0	0	0	0	67	0	0	0	0	0	0	19	0	1
13	0	0	0	0	0	0	0	0	0	1	0	0	0	0	0	0	0	0	0	0	0	0	21	0	1
0	0	13	0	0	0	0	0	0	0	0	0	0	1	0	0	0	0	0	0	0	0	0	27	0	2
0	0	0	0	0	0	0	1	0	0	1	0	1	0	1	1	0	0	0	0	0	0	0	33	0	3
0	0	0	0	0	0	0	1	0	1	1	1	1	0	1	1	0	0	0	0	0	0	0	39	0	5
0	0	0	0	0	1	0	1	0	0	1	1	0	0	1	1	0	0	0	0	0	0	0	21	0	6
0	0	0	0	0	1	1	1	0	1	2	1	0	0	1	0	0	0	0	0	1	0	0	21	0	5
0	0	0	0	1	0	3	0	0	1	0	0	0	0	1	1	26	0	0	0	1	0	0	21	0	4
0	0	0	0	1	2	1	1	0	1	1	0	1	0	3	0	25	0	0	0	1	0	0	21	0	4
12	0	0	0	1	1	2	1	1	1	0	1	0	0	1	0	25	0	0	0	1	0	0	21	0	5
24	0	0	0	3	2	3	1	1	6	3	2	0	0	3	0	25	1	0	0	6	5	0	31	37	7
12	2	24	0	14	38	15	8	6	36	15	26	9	2	28	3	37	4	1	0	11	47	0	20	76	19
70	10	54	0	100	65	62	67	100	95	100	80	96	64	55	24	75	51	8	32	64	78	0	100	85	74
43	12	87	0	90	100	100	100	75	100	71	100	100	100	100	48	100	93	34	100	100	100	0	73	53	100
50	4	38	0	38	27	35	25	38	43	33	36	72	31	40	15	64	31	10	30	35	42	0	39	100	48
22	3	21	0	25	14	24	17	16	26	19	24	35	15	24	9	43	24	10	39	13	29	58	27	99	33
40	2	16	0	19	7	21	10	9	15	15	14	19	9	19	5	42	14	5	20	16	13	57	35	63	27
11	1	22	0	14	6	22	8	17	15	12	11	20	7	14	4	31	10	5	20	9	8	58	44	81	23
22	1	10	0	12	5	14	9	7	14	8	11	16	7	14	4	40	32	9	19	10	14	59	92	32	20
10	1	10	0	9	4	10	6	5	9	6	24	11	5	12	3	40	13	4	37	10	10	62	29	48	16
10	1	22	0	9	4	8	5	5	7	5	7	11	4	9	2	41	9	33	38	35	10	50	29	49	13
10	1	11	0	8	5	7	5	4	8	5	5	9	4	7	2	31	5	17	38	11	14	50	100	49	14
10	1	11	0	7	3	6	4	4	7	4	5	8	3	10	2	21	4	100	18	7	21	50	28	39	14
10	1	11	0	14	4	6	5	5	9	4	7	10	4	9	2	31	6	39	19	7	18	55	28	31	19
10	1	24	100	11	28	6	5	4	7	3	4	9	4	7	3	41	3	18	18	11	16	47	45	30	14
11	1	14	0	13	9	8	4	3	10	4	4	9	3	7	2	30	6	12	18	4	12	47	17	31	12
12	1	13	0	7	7	8	3	4	7	4	16	7	3	6	2	31	5	7	18	11	16	47	19	31	12
12	5	12	0	9	5	7	6	4	8	3	6	8	3	5	3	31	5	9	19	14	13	0	25	32	12
12	3	12	0	10	4	8	6	4	10	11	8	7	3	7	6	21	5	4	19	8	17	0	32	63	14
22	1	16	0	9	4	7	9	7	7	5	6	9	3	7	2	31	3	3	23	9	10	0	38	94	14
31	1	20	0	11	4	6	10	6	8	5	34	13	3	10	3	40	4	3	26	10	12	0	77	32	17
12	1	11	72	8	3	7	6	6	5	4	19	8	1	12	2	32	4	3	30	6	8	0	18	31	13
22	1	11	0	6	2	6	4	3	5	3	10	10	3	8	2	32	3	2	33	5	8	0	18	31	11
10	1	11	0	9	2	7	5	4	6	2	9	12	4	19	2	83	2	2	37	6	12	0	18	30	12
11	1	23	0	9	2	6	4	4	6	3	12	20	2	12	2	20	4	2	0	5	19	0	19	31	12
11	1	34	0	8	2	6	4	6	6	2	9	10	2	11	1	20	3	1	0	7	8	44	19	32	11
11	1	11	0	9	2	6	5	5	11	3	7	8	1	7	1	22	3	2	0	9	16	100	30	32	11
22	0	12	0	10	2	8	6	9	11	2	6	9	1	5	1	31	3	1	0	5	14	45	20	32	11
11	1	15	0	12	2	19	11	9	18	4	20	13	1	8	1	41	4	2	0	6	4	45	48	31	16
18	1	18	0	27	4	13	20	9	18	6	13	20	2	14	2	20	5	6	41	7	16	45	19	31	30
11	1	22	0	16	3	8	8	5	9	3	9	13	2	10	2	23	4	2	44	4	16	45	24	32	19
40	1	12	0	14	3	7	12	9	13	2	7	12	1	10	2	32	3	1	48	5	4	45	29	32	17
47	4	26	0	13	3	9	9	9	10	3	7	13	1	8	2	41	5	1	51	2	8	45	34	32	18
30	100	40	0	12	3	9	9	8	9	2	7	16	91	56	100	29	47	7	55	21	63	45	18	31	25
67	36	42	63	11	3	6	8	5	7	4	4	12	33	57	65	20	100	9	59	18	50	46	18	31	18
100	17	61	67	9	1	4	6	4	6	2	5	11	14	66	38	20	37	9	23	9	46	67	22	31	13
49	10	100	70	7	2	4	5	3	4	2	3	5	9	28	22	20	33	8	23	3	28	89	27	31	10
63	7	95	0	7	2	4	5	2	3	2	3	5	8	27	15	20	36	4	23	8	15	46	32	31	9
12	4	47	0	7	1	3	4	2	4	2	3	3	5	26	13	21	23	4	19	3	20	0	36	31	7
11	3	78	0	6	1	3	4	2	4	1	2	6	5	23	10	22	33	7	19	4	25	0	18	31	7
12	2	60	0	5	1	3	4	1	3	1	2	4	4	10	7	21	21	22	0	4	10	0	18	45	7
16	2	59	0	6	1	3	4	2	3	2	2	5	3	12	5	21	10	9	0	7	12	0	19	0	6
29	2	33	61	7	1	3	4	1	4	1	2	3	3	11	4	0	9	30	0	3	9	0	19	0	6
