# Supplementary material for: Global reaction to the recent outbreaks of Zika virus: Insights from a Big Data analysis
Source: PLoS One. 2017 Sep 21;12(9):e0185263. doi: 10.1371/journal.pone.0185263 (PMC5608413; doi:10.1371/journal.pone.0185263)
Supplement: S2 Table — (PDF) [file pone.0185263.s002.pdf]

Country	RSV	Microcephaly	Suspected autochthonous cases	Confirmed autochthonous cases	Incidence rate (per 100,000 inhabitants)	Imported cases	Population	HDI
Guadeloupe	100	0	30775	379	6614,44	0	471000	0,841
Jamaica	86	0	6449	122	234,43	0	2803000	0,719
Trinidad & Tobago	76	1	0	643	47,11	1	1365000	0,772
Puerto Rico	60	3	0	31464	854,77	1	3681000	0,838
Honduras	57	1	31719	285	390,77	0	8190000	0,606
Dominican Republic	48	10	4884	328	48,94	0	10649	0,715
Nicaragua	44	0	0	2023	32,89	3	6150000	0,631
Venezuela	43	0	58758	2244	193,54	0	31519000	0,762
Guatemala	27	15	2785	466	19,5	0	16673000	0,627
Brazil	25	2079	200465	109596	147,95	0	209568000	0,755
Panama	24	5	1959	457	60,55	42	3990000	0,78
Costa Rica	23	1	2450	1355	78,34	32	4857000	0,766
El Salvador	23	4	11305	51	184,77	0	6146000	0,666
Colombia	22	54	95929	8826	215,31	0	48654000	0,72
Bolivia	19	3	597	128	6,6	4	10888000	0,662
United States	18	30	0	140	0,04	3988	324119000	0,915
Mexico	16	0	0	5854	4,55	15	128632000	0,756
Canada	15	1	0	0	0	359	36286000	0,913
Paraguay	15	2	546	12	8,3	0	6725000	0,679
Ecuador	12	0	2722	806	21,53	15	16385000	0,732
Uruguay	12	0	0	0	0	1	344000	0,793
Chile	9	0	0	0	0	29	18132000	0,832
Peru	9	0	0	124	0,4	17	31374000	0,734
Argentina	8	0	1821	26	4,21	27	43847000	0,836
Anguilla	0	0	38	5	252,94	1	17000	0,809
Antigua and Barbuda	0	0	393	14	432,98	2	94000	0,783
Aruba	0	0	614	28	563,16	7	114000	0,839
Bahamas	0	0	0	16	4,07	2	393000	0,79
Barbados	0	0	625	30	255,09	0	291000	0,785
Belize	0	0	537	49	159,67	0	367000	0,715
Bermuda	0	0	0	0	0	5	71000	0,901
Bonaire, St Eustatius and Saba	0	0	0	85	340	0	25000	0,823
Cayman Islands	0	0	201	29	403,51	10	57000	0,887
Curacao	0	0	0	322	216,11	0	149000	0,825
Cuba	0	0	0	3	0,03	30	11393000	0,769
Dominica	0	0	1150	79	1660,81	0	74000	0,724
French Guiana	0	10	9940	483	3776,45	10	276000	0,816
Grenada	0	1	313	100	372,07	0	11000	0,75
Guyana	0	0	0	6	0,78	0	771000	0,636
Haiti	0	1	2955	5	27,29	0	10848000	0,483
Martinique	0	12	36590	12	9242,93	0	396000	0,884
Montserrat	0	0	0	0	0	0	5000	0,731
Saint Barthelemy	0	0	820	61	9788,89	0	9000	0,876
Saint Kitts and Nevis	0	0	472	17	940,38	0	52000	0,752
Saint Lucia	0	0	822	50	531,71	0	164000	0,729
Saint Martin	0	0	2670	200	7972,22	0	36000	0,821
Saint Vincenti and the Grenadines	0	0	156	38	190,2	0	102000	0,72
Sint Maarten	0	0	168	62	560,98	0	41000	0,83
Suriname	0	2	2751	723	633,94	0	548000	0,714
Turks and Caicos Islands	0	0	115	12	249,02	3	51000	0,826
United Kingdom Virgin Islands	0	0	51	38	261,76	0	34000	0,808
United States Virgin Islands	0	0	803	462	1228,16	0	103000	0,851
