# Supplementary figures and images for: Global reaction to the recent outbreaks of Zika virus: Insights from a Big Data analysis
Source: PLoS One. 2017 Sep 21;12(9):e0185263. doi: 10.1371/journal.pone.0185263 (PMC5608413; doi:10.1371/journal.pone.0185263)

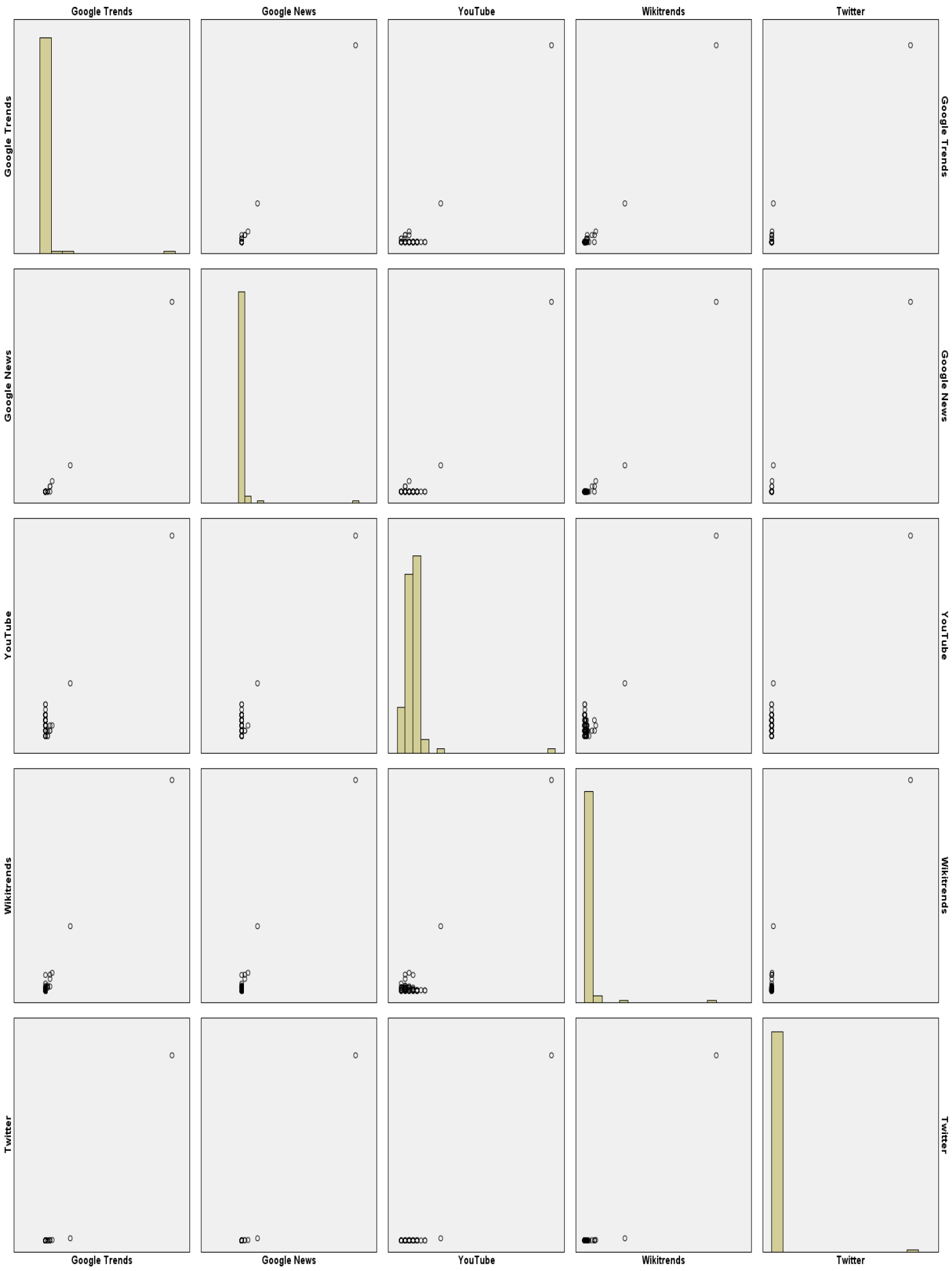

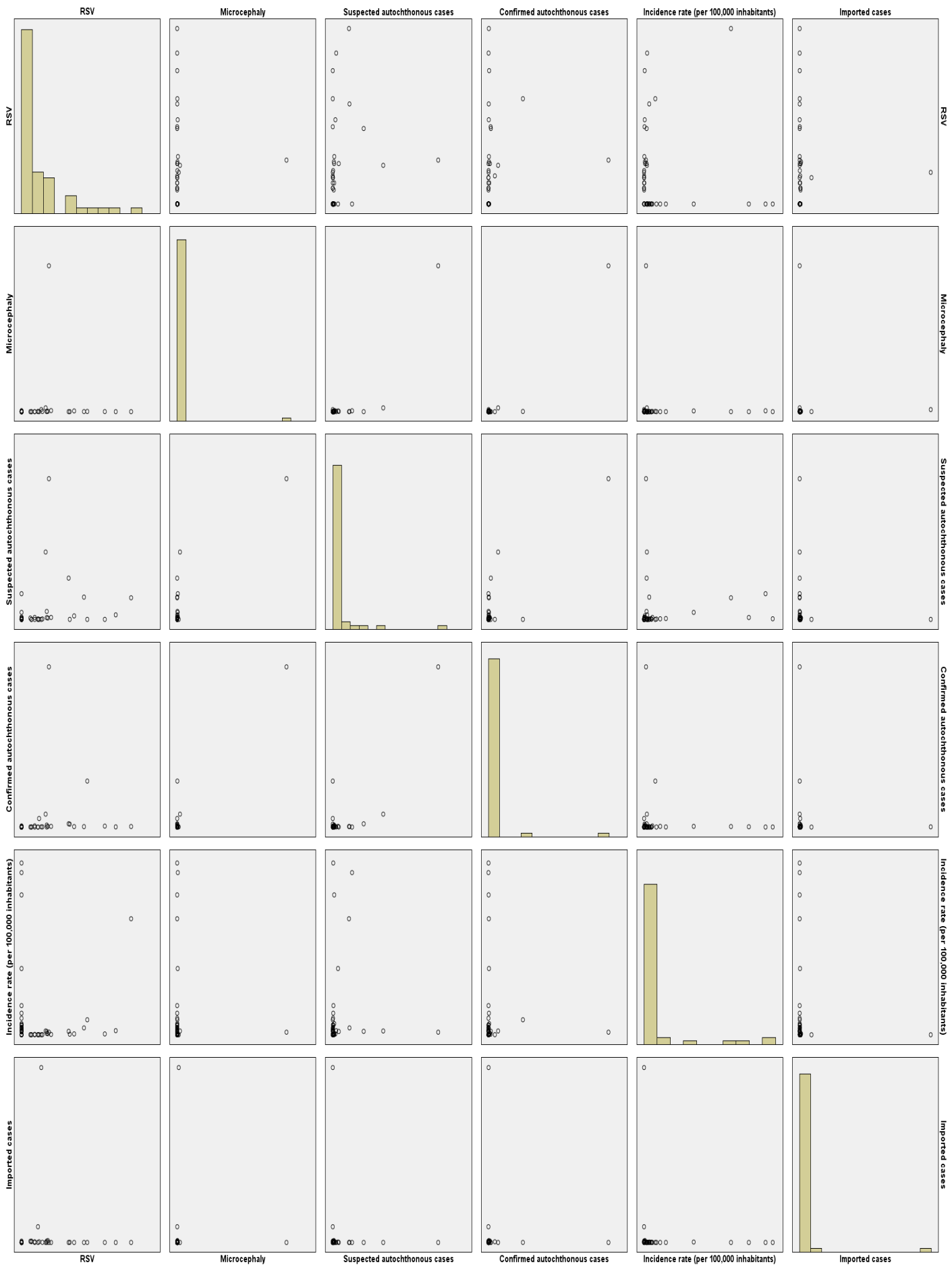

Supplement: S1 File — (PDF) [file pone.0185263.s003.pdf]
